# Behavior of Inflatable Drop-Stitch Fabric Panels Subjected to Bending and Compression

**DOI:** 10.3390/ma16216919

**Published:** 2023-10-28

**Authors:** William G. Davids

**Affiliations:** Department of Civil and Environmental Engineering, University of Maine, Orono, ME 04469-5711, USA; william.davids@maine.edu

**Keywords:** nonlinear finite element analysis, beam-columns, stability, post-buckling response, rapidly deployable shelters

## Abstract

In this paper, the mechanics of inflatable drop-stitch panels are investigated, including the impact of large shear deformations, nonlinearity due to wrinkling of the panel skin that occurs under net compressive strain, work done by the confined internal air, and the effect of the drop-stitch yarns on the panel skin stresses. A large deflection finite element (FE) analysis framework is presented that allows for a panel’s stability and post-buckling response to be quantified. The FE code is verified through comparison with available analytical solutions, and the impact of critical response drivers is examined. The FE models are then used to explore the capacity of panel walls when used as part of a shelter subject to realistic wind and snow loads and to assess the dependence of the capacity on the important design parameters of inflation pressure and panel depth. The analyses indicate that while the drop-stitch panel capacity is sensitive to the panel depth and inflation pressure, panels with reasonable cross-sectional dimensions are viable for use in structural applications where they must support significant compression and bending. Future work should focus on increasing the structural efficiency and capacity by increasing the panel shear stiffness and operational inflation pressure.

## 1. Introduction

**Figure 1:**
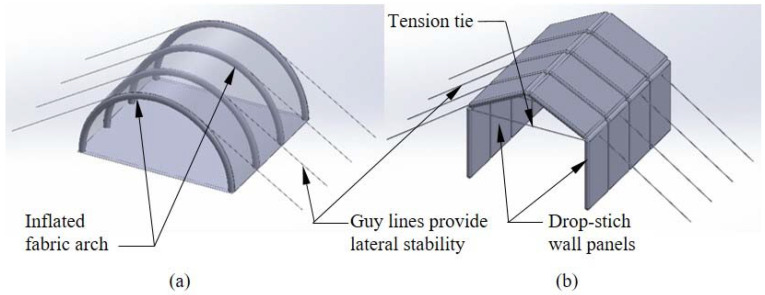
(**a**) Inflatable arch-supported structure; (**b**) proposed inflatable structure supported with drop-stitch panels.

Inflatable fabric beams, arches, and panels represent an important class of thin-walled structural members. Several currently deployed inflatable structures utilize parallel, regularly spaced inflated fabric arches with circular cross-sections that are covered by a fabric skin [1,2,3], and there are multiple commercial producers of such structures (Figure 1a). Compared to conventional rigid structures, they are exceptionally lightweight, regain their original shape and capacity following overloading, and can be stowed in a small volume when deflated and re-deployed multiple times.

Understanding the bending behavior of inflated members has long been recognized as essential for structural applications, and early analytical research focused on the response of inflatable beams with circular cross-sections defined by isotropic membranes [4,5,6,7]. Subsequent studies have numerically or analytically examined inflatable fabric beams [8,9,10,11], and experimental research has also been conducted on this topic [12,13]. One important response driver not considered in many of these studies is fabric wrinkling, a local buckling of the fabric shell that occurs when the stresses due to applied loads exceeds the initial prestress caused by inflation pressure and, in particular, the prediction of post-wrinkling behavior. Other studies have computationally assessed the loss of beam stiffness resulting from wrinkling using finite element (FE) analysis [14] or both computationally and experimentally [15,16,17]. Another important response driver is the stiffening behavior driven by the internal pressurized air as the beam undergoes deformation-induced volume changes. Fichter [7] first addressed this for shear deformations of a linearly elastic membrane by treating inflation pressure as a follower force. Over 40 years later, his original result was duplicated by the direct application pressure–volume work of the enclosed internal air (p−V work) within a Timoshenko beam framework, and extended to incorporate p−V work due to bending in the post-wrinkling range [14]. Experiments in which inflated fabric beams were subjected to large deflections have indicated the significance of pressure–volume work in both shear and bending [15]. While most prior studies have focused on the bending load–deflection response, a few have also addressed linearized buckling of straight inflated fabric columns without wrinkling [7,18] or a limited consideration of the buckling of straight inflated fabric columns accounting for fabric wrinkling [15]. While one recent study [19] has questioned the appropriateness of beam theory for the analysis of inflated fabric members, it is generally regarded as a good descriptor of their behavior and continues to be used by many researchers.

A smaller number of studies have considered the response of inflated fabric arches used in structures like the one shown in Figure 1a, which must carry both compression and bending caused by wind and snow loads. Arch stability and buckling were treated in [20], although the fabric wrinkling and the p−V work were not addressed in the study. The response of an entire structure with supporting arches and coupling beams was considered numerically and experimentally in [2], and the deployment of an inflated arch-supported structure was examined in [3]. A detailed experimental and computational treatment of the nonlinear response and the stability of the single arches can be found in [1], in which the techniques developed in [14,15] for the treatment of the wrinkling and the consideration of the p−V work were extended to address the combined compression and bending of the arches. In Reference [21], the impact of the wrinkling on the arch load–deformation response and the stability were addressed experimentally and computationally.

As a whole, these studies have provided significant insight into the response of inflated fabric arches and soft-wall arch-supported structures. However, for the general type of structure shown in Figure 1a, the fabric skin supported by the arches provides virtually no thermal insulation, which can necessitate the installation of insulating material on the inside of the structure. Further, the fabric skin is a flexible membrane prone to damage from high wind or debris, and it does not provide the occupants with the security inherent to a structure with stiffer, more resilient walls. Finally, the curved interior surface defined by the arches results in a high enclosed volume relative to the usable floor space, decreasing the efficiency.

**Figure 2:**
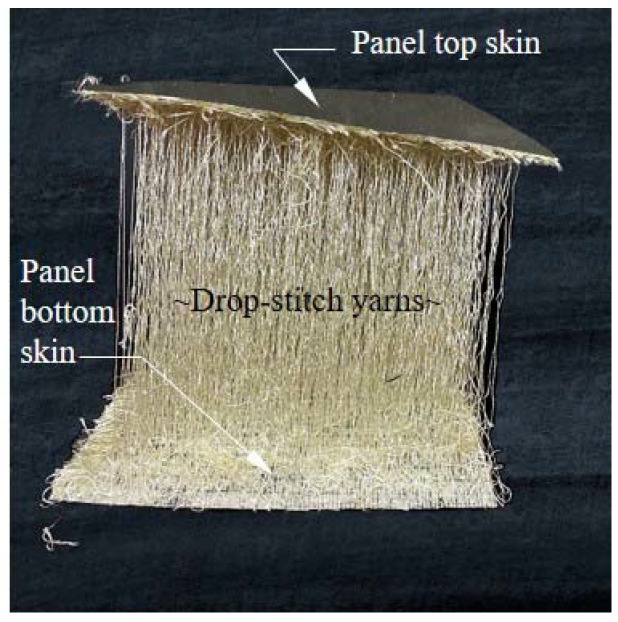
Image of a swatch of drop-stich fabric.

These observations lead to the consideration of the alternative lightweight, inflatable fabric structural configuration shown in Figure 1b. Unlike arches, this structure relies on vertical walls and roof members made from drop-stitch panels, which are inflatable fabric members distinct from more common inflated beams and arches that have circular cross-sections. In a drop-stich panel, yarns or threads of a fixed length are stitched to the upper and lower panel skins, as shown in Figure 2, with their length chosen to maintain a constant distance between the skins. When the edges and ends of the top and bottom skins are sealed, this creates an inflatable panel with flat top and bottom surfaces that are suitable for carrying directly applied transverse pressures caused by wind, snow, or other sources, ultimately making the geometry shown in Figure 1b feasible. Compared to the arch-supported structure in Figure 1a, the drop-stitch panels provide significant thermal insulation due to the air gap between the skins, and they are much stiffer than a fabric shell and lead to more efficient space utilization due to the vertical walls. However, the structural analysis and design of a shelter such as that shown in Figure 1b require a thorough understanding of the response of drop-stitch panels under bending, compression, and combined compression and bending.

Compared with inflated fabric beams and arches with circular cross-sections, drop-stitch panels have been the subject of relatively few studies. An early investigation [22] considered the experimental and numerical assessment of drop-stitch panel bending performance, demonstrating a pressure-dependent response. While the analyses in [22] did incorporate the impact of inflation pressure on shear deformations, they did not consider the post-wrinkling response and the nonlinearities caused by wrinkling, and only small panel deflections of ~5% of the simple span length were simulated. Reference [23] reports tests on several drop-stitch panels with three-point bending in which pressure-dependent response and fabric wrinkling were observed, but no significant analytical or computational predictions of the response were made. In [24], a drop-stitch panel was tested with four-point bending, showing an increasing stiffness and capacity with an increasing load and a significant panel post-wrinkling stiffness and capacity. However, the p−V work was considered in a limited manner, focusing only on the shear deformation, and the load–deflection response was only predicted in the linear, prewrinkling range. Some of the shortcomings of these studies were addressed in [25], in which a comprehensive experimental and numerical study considered the wrinkling, post-wrinkling response, p−V work, and the impact of drop-stitch yarns on the bending behavior. Most recently, analytical and FE-based solutions were developed for panels subjected to two-way bending, but only in the prewrinkling range [26]. However, no previous studies have considered the impact of compression and the combined compression and bending of drop-stitch panels, which is essential to quantifying the wall load-bearing capacity for the structural configuration shown in Figure 1b. Further, the impact of the drop-stitch yarns on panels subjected to combined compression and bending is unknown but potentially significant; this phenomenon is not relevant to the combined bending and compression of conventional inflated fabric beams and, thus, has not been previously considered for such members.

This study directly addressed this knowledge gap through the presentation and implementation of efficient, beam-element-based FE techniques specific to drop-stitch panels subjected to combined compression and bending in a large deflection framework. These methods allow for the direct incorporation of second-order bending effects, buckling stability, and the realistic prediction of post-buckling response. Beyond addressing geometric nonlinearities associated with global compressive buckling stability of drop-stitch panels, the methods presented here incorporate the local buckling (wrinkling) of the thin fabric skin that occurs when inflation-induced prestress is overcome by compressive stresses due to applied loads. This wrinkling is a significant source of nonlinearity that can impact both panel bending stiffness and stability. A suite of FE simulations was conducted to explore the relative significance of the important response drivers on panel bending and buckling response. Finally, FE techniques were used in a parametric study to explore the design space for the structural configuration shown in Figure 1b under the action of realistic wind and snow loads. This research is novel in its consideration of the combined compression and bending of drop-stitch panels and application to the capacity analysis of a practical structural configuration under realistic loading.

## 2. Mechanics of Drop-Stitch Panels in Bending and Compression

**Figure 3:**
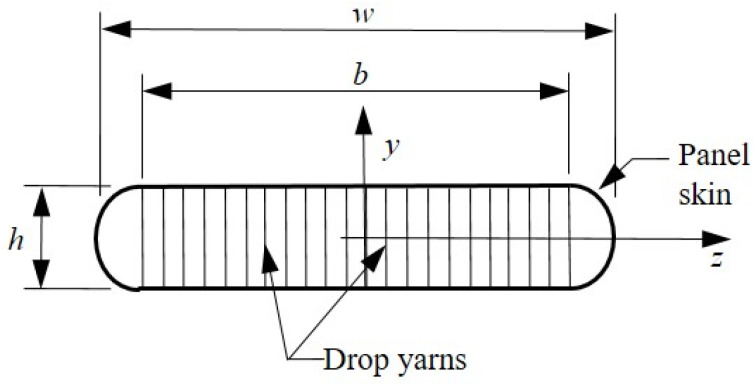
Schematic of the drop-stitch panel cross-section and definition of the panel geometric parameters.

When simulating the load–deformation, buckling and post-buckling behavior of drop-stitch panels subjected to bending and compression, it is essential that the following response drivers be captured: (1) inability of the fabric to carry compressive stress (wrinkling); (2) work done by the internal pressurized air due to deformation-induced volume changes; and (3) the effect of the drop-stitch yarns themselves on the stresses in the panel skin. Each of these response drivers is impacted by parameters such as panel inflation pressure, p, and the geometry of the drop-stitch panel defined in Figure 3. The description of each of these response drivers is brief, and the reader is directed to the references provided within for additional details as needed.

### 2.1. Wrinkling of the Panel Skin

Wrinkling occurs when the compressive stress due to external loads exceeds the initial tensile prestress caused by inflation. Equation (1) provided below defines the wrinkling bending moment, $M_{w}$, for a panel subjected to an external axial force, F (compression negative), and bent about the *z*-axis. In Equation (1), $I_{zz}$ is the moment of inertia (second moment of area) of the panel, $A_{s}$ is the perimeter defined by the fabric skin, $A_{p}=\frac{\pi h^{2}}{4}+bh$ is the cross-sectional area of the entire panel subjected to the inflation pressure p, and $P=pA_{p}$ is the axial pressure resultant. Throughout this paper, the panel skin thickness was considered to be small and incorporated into the membrane elastic moduli E and G, which both have units of force/length, as discussed later; therefore, $A_{s}=2b+\pi h$ and Izz=πr2+2bh22.

(1) $$M_{w}=\frac{2I_{zz}}{hA_{s}}\left(P+F\right)$$

As shown in [25], this value of $M_{w}$ overestimates the actual moment of the onset of wrinkling due to the interaction of the drop-stitch yarns with the panel skins. This topic is addressed later in this section.

The panel skin is assumed to be linearly elastic in tension with an effective axial membrane modulus E that is the product of the usual elastic modulus and the panel skin thickness. The linearly elastic behavior of the panel skin in tension has been experimentally shown to reasonably approximate the fabric skin response over the range of stresses typically experienced by a panel [25], and the vast majority of prior research assumes linearly elastic fabric response prewrinkling. To capture the effect of wrinkling, portions of the panel skin that experience a net compressive strain are assigned a modulus E=0 to reflect the inability of the fabric to carry compressive stress. This treatment of the panel skin as a bilinear, tension-only material results in a materially nonlinear moment–curvature response when the panel is subjected to bending about the *z*-axis. This nonlinear moment–curvature relationship is numerically generated for a given panel geometry, inflation pressure, and axial load F through a two-step process. First, the section’s neutral axis location is determined by enforcing the horizontal force equilibrium for the panel cross-section and a specified bending curvature κ. Then, the internal bending moment corresponding to that curvature is determined by numerically computing the integral shown in Equation (2), where σ is the *x*-direction stress in the panel skin at any point in the cross-section, and y is as defined in Figure 3.

(2) $$M=\int \sigma ydA_{s}$$

This process is repeated for a range of curvatures to define the complete M−κ relationship corresponding to a specific axial load, F. The numerical nonlinear moment–curvature analysis overviewed here has been performed for inflated fabric arches with circular cross-sections that carry combined bending and compression [1] and for drop-stitch panels under only bending (i.e., F=0) [25], and further details can be found in these references.

**Figure 4:**
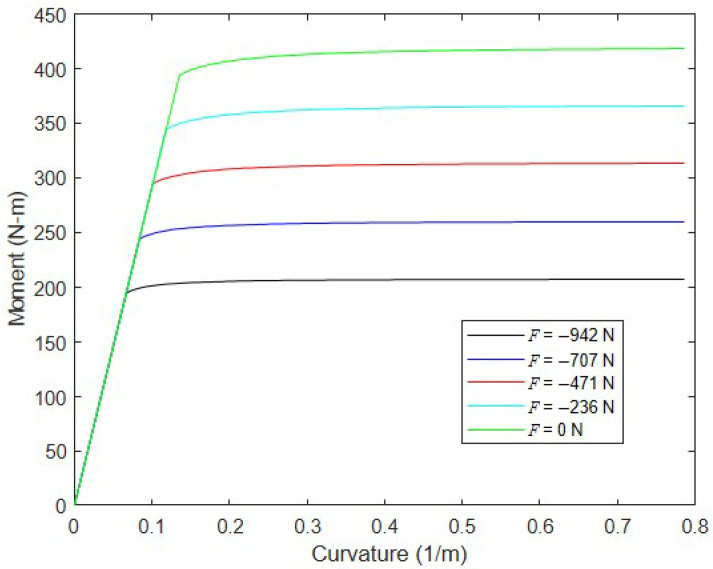
Moment–curvature relationships for a typical panel with different levels of applied compressive force.

To illustrate, Figure 4 shows the moment–curvature response of a drop-stitch panel with h = 102 mm, w = 1219 mm, p = 68.9 kPa, and E=472 N/mm for several values of axial loads F over the range (−F2, 0). This value of E is not arbitrary but is based on experiments of the drop-stitch panel fabric reported in Reference [25]. As expected, the onset of wrinkling leads to an abrupt loss of panel bending stiffness, since the entire top skin width b loses tension at the same time and wrinkling quickly progresses down the semicircular panel sidewalls. The application of significant compression forces hastens the onset of wrinkling as predicted by Equation (1). For reference, Equation (1) predicts $M_{w}$ = 402 N-m for the given values assuming F = 0, which agrees very well with the results shown in Figure 4.

### 2.2. Pressure–Volume Work

**Figure 5:**
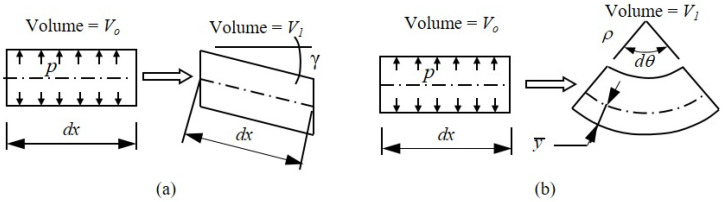
Volume changes due to (**a**) shear deformation and (**b**) post-wrinkling bending.

Prior research has shown that incorporating the pressure–volume (p−V) work done by the confined, pressurized internal air has a significant impact on the bending and shear stiffness of the inflated fabric beams and arches [1,15]. Figure 5 illustrates the volume changes caused by an engineering shear strain, γ, and the bending rotation, dθ=κdx, of an inflated panel, where Timoshenko beam theory is used to define the shear deformation.

Considering the p−V work within a virtual work framework has shown that the work done by the internal pressure as the air volume decreases because of shear deformations gives rise to an additional shearing rigidity equal to the pressure resultant P [14]. As a result, the effective shear rigidity of an inflated beam is the sum $GA_{v}+P$, where G is the fabric shear modulus, and $A_{v}$ is the shear area. This is identical to the result derived by Fichter [7], who first studied the bending and buckling of inflated beams and columns with circular cross-sections made from shear-deformable membranes, in which pressure was treated as a follower force. For the drop-stitch panels considered here, shear stress was carried almost entirely by the semicircular side walls; applying the usual shear form factor of 2 for a circular section results in $A_{v}=\pi \frac{h}{2}$, where G is the membrane shear modulus with units of force per length. Recent experimental and computational research on the load–deformation response of drop-stitch panels under four-point bending has confirmed the large shear deformations and the importance of including the effect of pressure on shear stiffness [25].

The inclusion of p−V work due to bending deformations is more complex, but it has been considered in past studies as well [14,15]. Unlike p−V work due to shear, bending p−V work only occurs after wrinkling, as the bending neutral axis $\bar{y}$ drops below the mid-height of the section, as shown in Figure 5b. Once wrinkling initiates, this leads to the additional internal bending moment of Equation (3), which was first shown in Reference [14], for members with circular sections.

(3) $$M=P\left(\frac{h}{2}-\bar{y}\right)$$

The inclusion of the additional moment given in Equation (3) in the moment–curvature relationship stiffens and strengthens the post-wrinkling response of the panel. Prior coupled experimental and computational work on the bending of both tubular inflated fabric members [15] and drop-stitch panels [25] has demonstrated that neglecting p−V work due to bending results in the underestimation of the capacity and post-wrinkling stiffness.

### 2.3. Effect of the Drop-Stitch Yarns on Stresses in the Panel Skin

**Figure 6:**
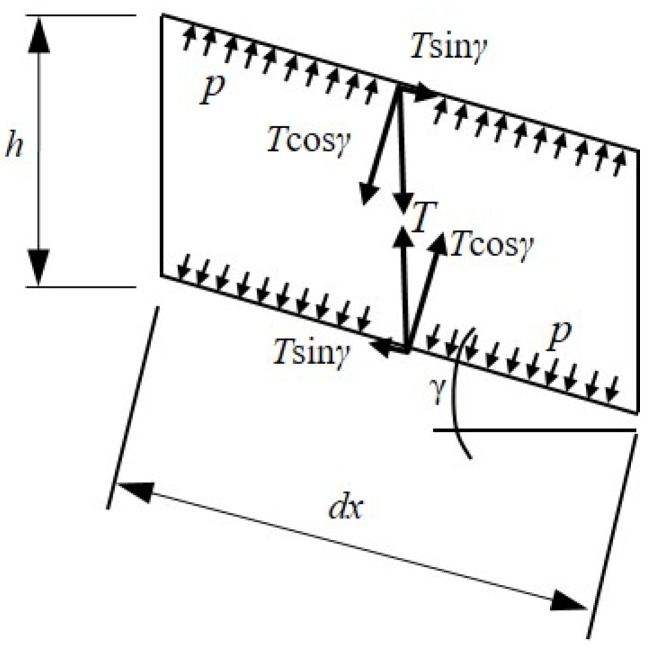
Short length of the drop-stitch panel undergoing shear deformation.

Drop-stitch yarns have also been shown to impact the panel bending response [25], and quantifying their effect requires considering the equilibrium of the panel in its deformed position. Figure 6 shows an elevated view of an infinitesimal length of panel, dx, subject to an engineering shear strain, γ, per Timoshenko beam theory, whereby the force, T, represents the tension in the drop-stitch yarns that are tributary to the length, dx, and width, b. In the deformed state, the drop-stitch yarns remain vertically oriented while the inflation pressure, p, acting on the top and bottom panel skins are perpendicular to the panel skins. The equilibrium then requires that Equation (4) [25] be satisfied.

(4) Tcos⁡γ=pbdx

However, the components Tsin⁡γ are equal and opposite forces which then form the distributed bending moment given by Equation (5). While Equation (5) was first derived in Reference [25], the small angle approximation γ≈sin⁡(γ) was employed. However, for the finite element development and simulations presented later in Section 3, Section 4 and Section 5 of this paper, this small angle approximation is not assumed.

(5) $$\frac{dM_{\gamma }}{dx}=pbh\sin\gamma$$

The distributed moment $\frac{dM_{\gamma }}{dx}$ tends to decrease the stress in the top skin of the panel and increase the stress in the bottom skin, effectively reducing the panel stiffness and the wrinkling moment.

**Figure 7:**
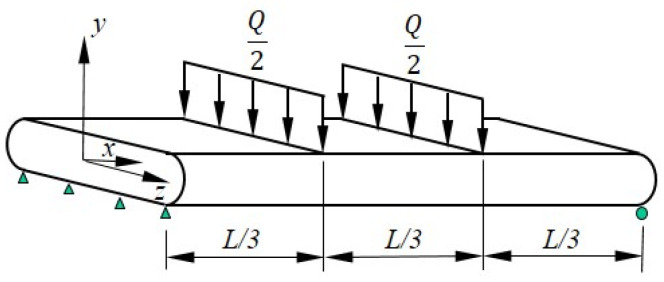
Drop-stitch panel loaded in bending.

To explore this further, consider a simply supported drop-stitch panel of length L subject to a transverse load Q applied at its third points and distributed evenly across the panel width, as shown in Figure 7. The constant shear strain in the end thirds of the span due to Q will cause a uniformly distributed moment at approximately the panel’s *z*-axis, as defined in Equation (5), which effectively increases the panel’s bending.

Considering the equilibrium of the unloaded, middle third of the panel implies it carries a total internal moment M, as defined in Equation (6). Setting this equal to the wrinkling moment of Equation (1) and assuming γ is small so that γ≈sin⁡(γ) allows the wrinkling load $Q_{w}$ to be computed explicitly as shown in Equation (7). (Note that if the approximation γ≈sin⁡(γ) is not used, Equation (6) can be solved numerically for $Q_{w}$; however, the small angle approximation is very accurate for the magnitudes of shear strains considered here.)

(6) M=QL6+∫0L/3pbhsin⁡Q2GAv+Pdx=QL6+pbhL3sin⁡Q2GAv+P

(7) Qw=12PIzzhAsL1+pbhGAv+P

Equation (7) shows that the wrinkling load $Q_{w}$ decreases as shear deformations increase. Similarly, the distributed moment $\frac{dM_{\gamma }}{dx}$ will increase the deflections. Considering only the load in Figure 7 and applying Timoshenko beam theory, the mid-span deflections of the beam at the onset of the wrinkling due to the bending ${∆}_{b}$ and shear ${∆}_{s}$ are provided in Equations (8) and (9), respectively.

(8) $${∆}_{b}=\frac{23Q_{w}L^{3}}{1296EI}$$

(9) $${∆}_{s}=\frac{Q_{w}L}{6\left(GA_{v}+P\right)}$$

The additional deflection ${∆}_{DS}$ caused by $\frac{dM_{\gamma }}{dx}$ is computed by adapting the less common equation for mid-span deflection due to a concentrated moment found in Reference [27], as shown in Equation (10).

(10) $${∆}_{DS}=2\int_{2L/3}^{L}\frac{\frac{dM_{\gamma }}{dx}}{12EI}\left(6xL-3x^{2}-\frac{9L^{2}}{4}\right)dx=\frac{pbh\sin\gamma }{6EI}\int_{2L/3}^{L}\left(6xL-3x^{2}-\frac{9L^{2}}{4}\right)dx=\frac{23L^{3}pbh\sin\gamma }{648EI}$$

**Figure 8:**
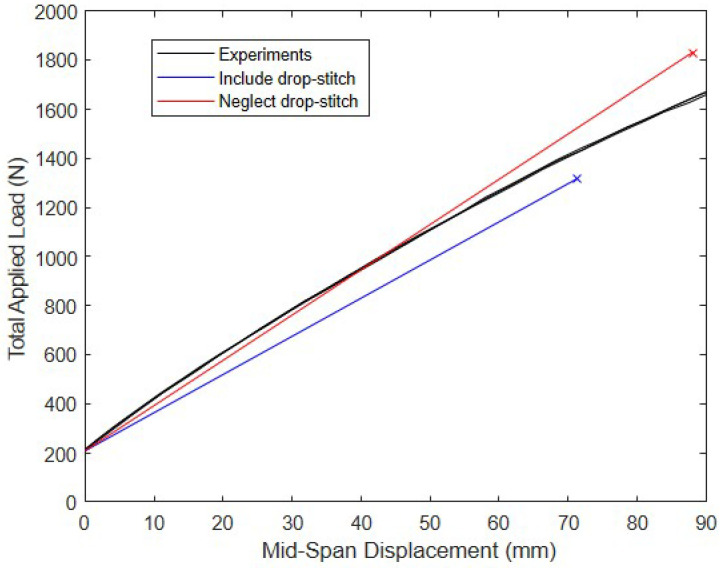
Experimental and predicted prewrinkling bending response of the panel tested in Reference [25].

To assess the effect of the drop-stitch yarns in a practical setting, the prewrinkling load–deflection response predicted using Equations (5)–(10) is compared with the measured load–deflection response of the 2.13 m span panel fabricated from 420 × 420 denier nylon drop-stitch fabric tested in Reference [25]. The panel was loaded in four-point bending, as shown in Figure 7, and had properties of b= 572 mm, h= 178 mm, and p= 68.9 kPa. The panel cross-sectional geometry was taken as defined in Figure 3. The drop-stitch fabric elastic and shear moduli E and G were 472 N/mm and 33.6 N/mm, respectively, as reported based on experiments detailed in Reference [25]. The results of the calculations and the experiment are given in Figure 8. The initial nonzero load of 209 N at zero displacement represents the weight of the loading fixture and was accounted for in all computed displacements. The bending test was run three times, and the results were very repeatable, as shown in Figure 8, which includes the results from all three replicates.

When the additional bending due to the drop-stitch yarn is considered, Equation (7) predicts $Q_{w}$ = 1319 N, which produces a mid-span displacement of ${∆}_{b}+{∆}_{s}+{∆}_{DS}=$ 71.4 mm. At the same displacement, the average experimentally measured load is 1427 N, a difference of 8.2%. In contrast, if the additional bending caused by the drop-stitch yarn is neglected, the computed wrinkling load increases to 1830 N at a displacement ${∆}_{b}+{∆}_{s}=$ 88.1 mm. This load of 1830 N exceeds the average experimental load of 1641 N corresponding to 88.1 mm by 11.5%. These results indicate that the inclusion of the additional moment caused by the drop-stitch yarns given in Equation (5) is important for the accurate prediction of the response. The significance of this and other factors in the post-wrinkling range is explored later in this paper.

## 3. FE Implementation

### 3.1. Virtual Work Framework

The starting point for the model’s implementation is the virtual work framework used previously for inflated fabric members with circular cross-sections [1,14]. One simplification is that the axial load–deformation response is considered linearly elastic, but all other essential elements of the response described in the previous section are captured. Given that axial deformations are typically small in columns and beam-columns, this is a reasonable simplification, and it has been used successfully in the past for the analysis of inflated tubular fabric arches [1]. The tensile responses of the panel skin in the bending and shear stress–strain responses are also considered linearly elastic, which prior experimental research has demonstrated to be reasonable for drop-stitch materials [25]. However, the virtual work formulation and numerically generated moment–curvature relationships employed here can easily accommodate material nonlinearity.

**Figure 9:**
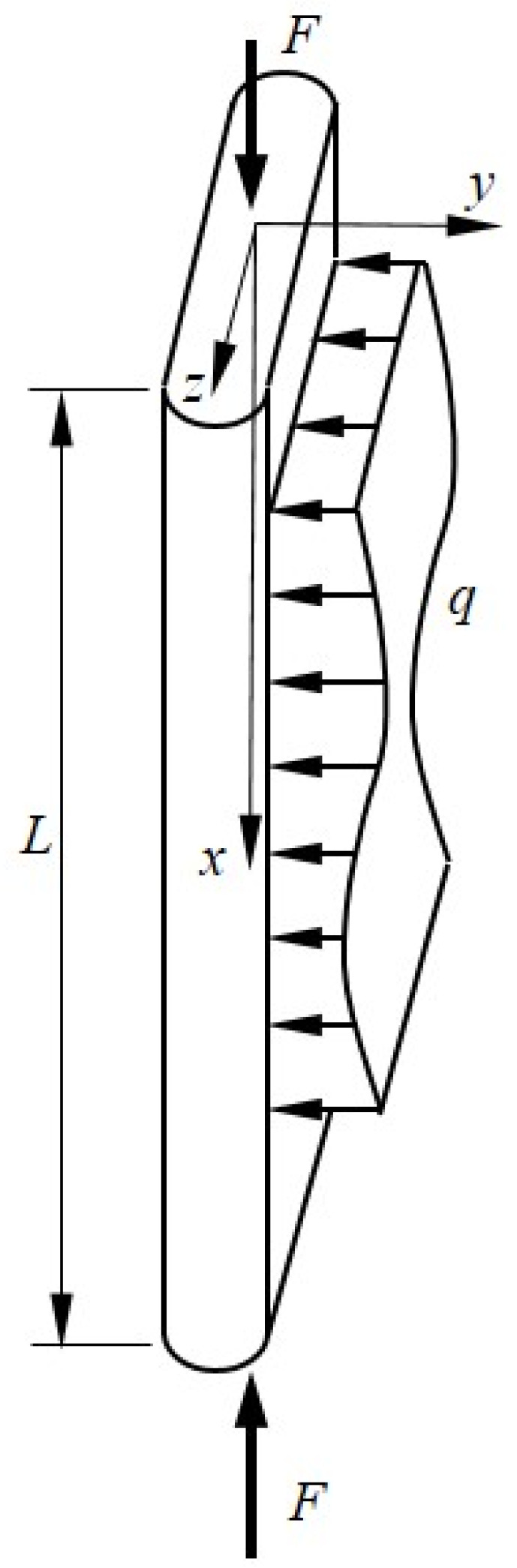
Drop-stitch panel under axial and transverse loads.

Figure 9 shows a typical drop-stitch panel of length *L* subject to transverse loads q and an axial force F. The transverse load q is uniformly distributed across the panel width and produces bending about the *z*-axis, and F is evenly distributed over the panel ends producing uniform compressive stress. The panel cross-sectional geometry is defined as shown previously in Figure 3. Assuming a nonlinear moment–curvature response as detailed in the last section, the internal virtual work $\delta W^{int}$ can be expressed as shown below in Equation (11) [1], where ϵ denotes the axial strain, κ is the bending curvature, and γ is the engineering shear strain. When preceded by δ, these are virtual quantities through which the corresponding real stress resultant acts.

(11) $$\delta W^{int}=\int EA_{p}\epsilon \delta \epsilon dx+\int \left(M+P\left(\frac{h}{2}-\bar{y}\right)\right)\kappa \delta \kappa dx+\int \left(GA_{s}+P\right)\gamma \delta \gamma$$

The pressure–volume work due to bending appears in the second integral through the inclusion of the additional moment given by Equation (3), and the pressure–volume work due to shear appears in the third integral where the pressure resultant P increases the effective shear rigidity.

The external virtual work is given by Equation (12) below. Here, the distributed moment produced by the drop-stitch yarns is treated as a follower force, the magnitude of which changes as the panel undergoes shear deformation.

(12) $$\delta W^{ext}=F\delta u+\int q\delta vdx+\int \left(\int pbh\sin\gamma dx\right)\delta \theta$$

Further, δu is a virtual axial displacement, δv is a virtual transverse displacement, and δθ is a virtual rotation. All virtual strains—δϵ,  δκ, and δγ—must be compatible with the corresponding virtual displacements and rotations. The inner integral of the final term on the right-hand side of Equation (12) captures the moment caused by the drop-stitch yarns, and its virtual work is computed as the moment acting through the virtual rotation. The final term can be written as shown in Equation (13) after applying the kinematic requirement that the bending rotation δθ=δκdx.

(13) $$\int \left(\int pbh\sin\gamma dx\right)\delta \theta =\int \left(\int pbh\sin\gamma dx\right)\delta \kappa dx$$

To capture the nonlinear moment response created by the wrinkling of the panel skin and the post-wrinkling p−V work, the effective internal moment, $M^{e}$, is defined in Equation (14) and expanded in the vicinity of the current displaced shape using a Taylor series approximation, as defined in Equation (15) [1,14].

(14) $$M^{e}=M+P\left(\frac{h}{2}-\bar{y}\right)$$

(15) $$M^{e}\left(\kappa +\Delta \kappa \right)=M^{e}+\frac{dM^{e}}{d\kappa }\Delta \kappa =M^{e}+\frac{dM}{d\kappa }\Delta \kappa -P\frac{d\bar{y}}{d\kappa }\Delta \kappa$$

An examination of Equation (15) shows that the nonlinear moment–curvature response and bending p−V work combine to result in the effective tangent bending rigidity ${EI}^{e}$, defined by Equation (16), which is easily computed via numerical differentiation at a given curvature [1,14].

(16) $${EI}^{e}=\frac{dM}{d\kappa }-P\frac{d\bar{y}}{d\kappa }$$

Simpler expansions can be made for the axial and shear terms due to their linear nature, and the incremental form of the virtual work expression can be written as shown in Equation (17) after equating the internal and external virtual work.

(17) $$\int EA_{p}\Delta \epsilon \delta \epsilon dx+\int {EI}^{e}\Delta \kappa \delta \kappa dx+\int \left(GA_{s}+P\right)\Delta \gamma \delta \gamma dx=F\delta u-\int q\delta vdx+\int \left(\int pbh\sin\gamma dx\right)\delta \kappa dx-\;\int EA\epsilon \delta \epsilon dx-\int \left(M+P\left(\frac{h}{2}-\bar{y}\right)\right)\delta \kappa dx-\int \left(GA_{v}+P\right)\gamma \delta \gamma dx$$

### 3.2. Finite Element Discretization and Solution

The incremental virtual work expression is discretized with a three-noded, Timoshenko beam element. The transverse nodal displacements vary quadratically along the element’s length, and the nodal rotations are specified to vary linearly to avoid shear locking. Details on this element and its shape functions can be found in [14]. To simplify the formulation for the present simulations, distributed loads were applied as point loads at the starting and ending nodes of each element, which maintained constant shear force and shear strain within each element. This allowed for not only the loading to be simplified but permitted the inner integral in Equation (13) to be easily evaluated for each element, since γ is constant within each element. This led to an applied bending moment at each end of every element that experienced shear deformation that was equal to $\frac{1}{2}pbh\sin\gamma l$, where l is the element length.

To assess the buckling and post-buckling response of the drop-stitch panels loaded in both bending and compression, a large deflection co-rotational analysis was implemented in which the equilibrium was satisfied in the element deformed position [28]. A co-rotational formulation is advantageous for such analyses because it treats the displacement of each element as the sum of the rigid-body and strain-producing displacements in the original coordinate system. The nodal locations were updated at every step in the solution process, and the usual element material tangent stiffness can be calculated in a straightforward manner, accounting for a materially nonlinear response in bending, and the additional necessary geometric stiffness matrix is well defined. More details on the application of this approach can be found in [1], which treats the analysis of inflatable fabric arches with circular cross-sections. The solver incorporates automatic load stepping with two solution algorithms. At the start of the analysis, Newton’s method is used. When the structure approaches a bifurcation point corresponding to the onset of buckling or other strong nonlinearity, Newton’s method typically fails to converge, and the solver switches automatically to Crisfield’s arc-length method [29]. This allows for the sharp nonlinearity due to an abrupt loss of the bending’s stiffness caused by the fabric wrinkling and post-buckling responses, including softening to be captured. All of the FE codes used in this study were implemented by the author in MATLAB.

### 3.3. Model Convergence and Verification

To assess the model convergence and verify the predictions, the buckling of a drop-stitch panel with L=2438 mm, h=102 mm, and w=1219 mm with pinned–pinned ends was simulated. The inflation pressure, p, was taken as 68.9 kPa, a typical operating pressure for such panels with corresponding material properties, E = 472 N/mm, and G = 33.6 N/mm. As discussed previously, these values for E and G were determined experimentally in Reference [25] for a drop-stitch panel made from woven nylon fabric, as shown in Figure 2, and realistically capture the small value of G relative to E that drives large shear deformations. Panel buckling was simulated by assigning the panel an initial shape corresponding to a ½ sin wave with a central perturbation of 2.5 mm (~0.01L) relative to the plumb, and the panel was subjected only to a compressive load of F. Simulations were run for mid-height transverse y-direction deflections up to 300 mm (12.3% of the panel height) to capture the large deformation post-buckling behavior.

**Figure 10:**
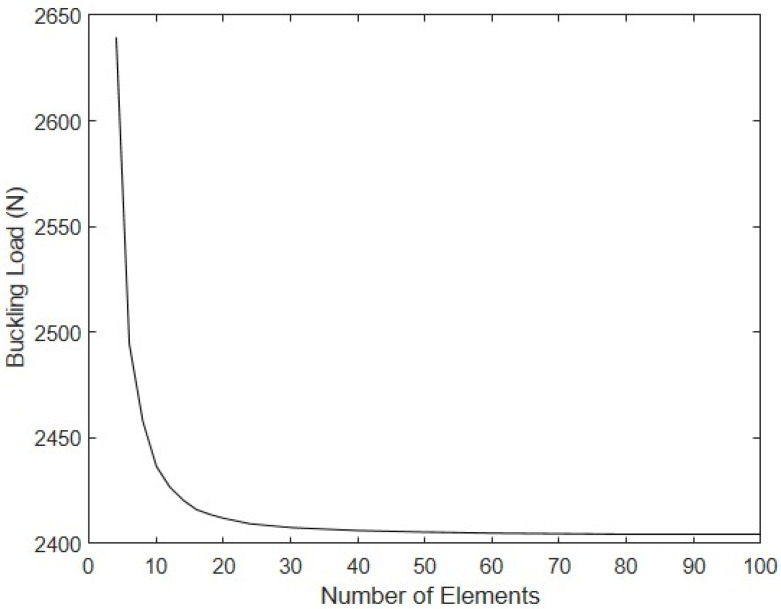
Buckling load vs. number of elements.

**Figure 11:**
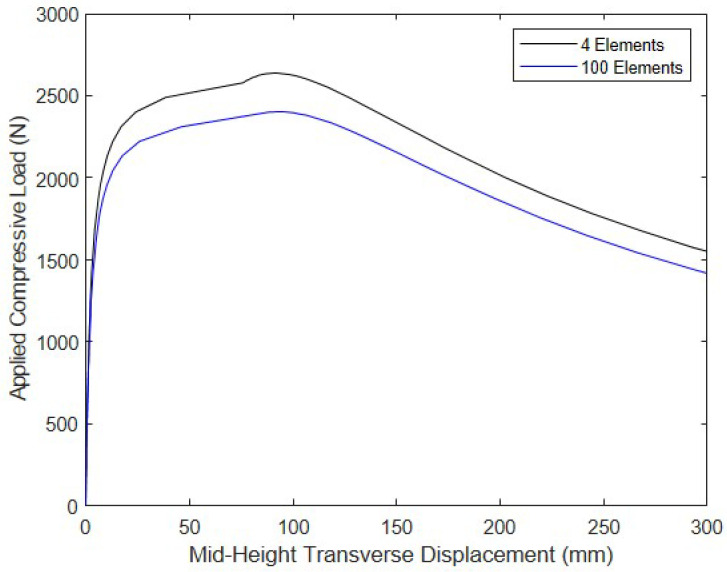
Axial compression vs. mid-height transverse displacement.

The buckling load vs. the number of equal-length elements used to discretize the drop-stitch column is shown in Figure 10. The results indicate that convergence was rapid and smooth, with the model reaching a value of 2405 N when 60 or more elements were used. To illustrate the buckling and post-buckling response at large deformations, Figure 11 shows the relationship between the mid-height transverse y-direction deflection and the magnitude of the applied compressive force for the coarsest mesh with 4 elements and the most refined mesh with 100 elements (the 60-element mesh led to results that were indistinguishable from the 100-element model). While the response is, of course, different for the two meshes, they both exhibited similar responses, as the buckling load approached and in the post-buckling regime. The small amount of stiffening that occurred at deflections of ~80–90 mm can be attributed to wrinkling that occurs after the initiation of significant transverse deflections, which is corroborated by the 100-element model predicting the onset of wrinkling when F = 2402 N at a corresponding transverse displacement of 89 mm. On the basis of this convergence study, all further simulations were conducted with the 60-element model.

To verify the FE formulation, its predictions can be compared with the critical membrane buckling stress, $\sigma_{cr}$, derived by Fichter [7] for a shear-deformable inflated column, as given in Equation (18).

(18) $$\sigma_{cr}=\frac{EI\frac{\pi^{2}}{L^{2}}\left(P+GA_{v}\right)}{EI\frac{\pi^{2}}{L^{2}}+P+GA_{v}}$$

Computing $\sigma_{cr}$ for the panel properties used here and multiplying the result by the perimeter of the cross-section $A_{p}$ results in a buckling load of 3581 N. This value is significantly greater than the FE-predicted value of 2405 N because of the FE model’s incorporation of the drop-stitch fabric and, to a lesser extent, panel wrinkling, neither of which were addressed in the development of Equation (18). Indeed, when the FE simulation is run without wrinkling and neglecting the drop-stitch yarns—accomplished by neglecting the distributed moment of Equation (5) in the simulation—the FE model predicts a buckling load of 3560 N, which differs by only 0.6% from the value predicted using Equation (18). This highlights the significance of the drop-stitch yarns on the response, which is explored further in the next section along with other response drivers, such as shear deformations, wrinkling, and panel inflation pressure.

## 4. Impact of Panel Characteristics and Transverse Loads on Response

### 4.1. Impact of Shear Deformations, Drop Yarns, and Wrinkling on Pure Compression

**Figure 12:**
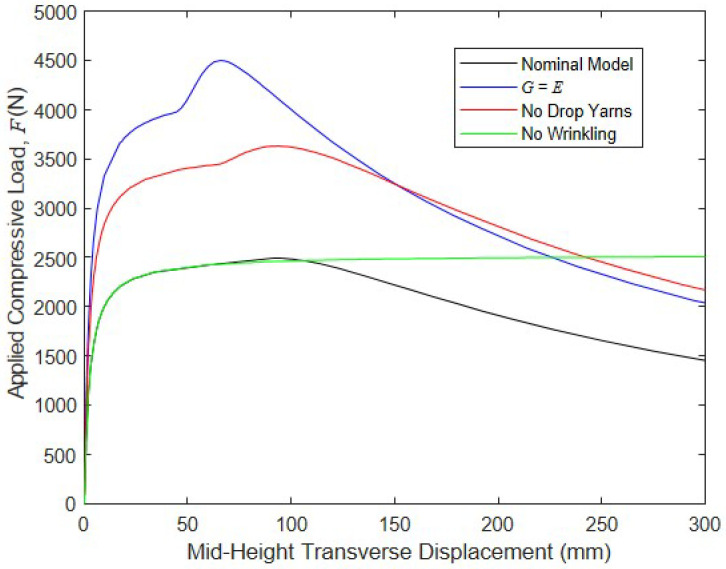
Impact of the parameters on the buckling and post-buckling responses.

To assess the impact of major contributors to global compressive buckling and post-buckling responses, the previously described FE model was run for cases including G=E, which makes the shear deformations small; drop yarns were excluded; and wrinkling was excluded. The results of these simulations are shown below in Figure 12, along with the results from the nominal model in which all of the response drivers were present. They highlight that shear deformation significantly decreased the panel’s compressive strength: as G grew from the experimentally determined value of 33.6 N/mm to 472 N/mm, the panel’s critical load increased by 81% to 4510 N. Further, since shear deformations are small when G=E, the additional moment caused by the drop-stitch yarns, as predicted by Equation (5), was negligible, which increased the buckling resistance of the panel compared to the nominal model. This allowed for the wrinkling and bending pressure–volume work to be mobilized prior to the global buckling at a displacement of approximately 50 mm, and the bending pressure–volume work, in turn, caused an increase in the stiffness prior to the peak load of 4510 N being reached at a displacement of approximately 70 mm. The drop-stitch yarns were also significant drivers of the response: when the effect of these yarns, as captured by Equation (5), was neglected, the panel buckling load increased by 46% relative to the nominal case. However, it is important to note that since the additional moment caused by the drop-stitch yarns was driven by shear deformation, increasing G to a large value greatly reduced the negative effect of the drop-stitch yarns on the buckling.

Neglecting the bending-induced wrinkling for this combination of parameters did not have a large effect on the critical load, although it significantly impacted the panel’s post-peak response. The nominal model with all factors considered experienced significant post-peak softening due to wrinkling, whereas the model without wrinkling remained linearly elastic and continued to gain axial capacity over the full 300 mm of transverse mid-height displacement considered here. A final simulation was run in which the p−V work in both the shear and bending was neglected, although the results are not shown in Figure 12, since the model was unable to converge after reaching a peak load of 1334 N, which was 46% less than the peak load carried by the nominal model, at a displacement of only 30 mm. This dramatic drop in capacity and the inability of the model to converge post-buckling is due to the very low shear stiffness when the p−V work was neglected, since the panel $GA_{v}=$ 5360 N is no longer increased by the pressure resultant P= 8410 N.

### 4.2. Impact of Transverse Load and Inflation Pressure

Drop-stitch panels that are used for the walls of a structure such as the one shown in Figure 1b are required to simultaneously support gravity loads (caused primarily by snow) and transverse loads (caused primarily by wind), making it necessary to assess the impact of combined vertical compression and transverse loading. Here, the same nominal model was analyzed under an increasing vertical compressive load F, while a constant transverse load Q= 328 N was applied at the wall mid-height. This transverse load produces 50% of the theoretical wrinkling moment for the panel when F=0 based on Equation (1). As a result, as the compression force, F, grows, second-order bending effects will increase the internal panel moment, causing wrinkling and softening the panel response. As with the buckling analyses presented previously, models were also run for cases in which G=E, drop yarns were excluded, and wrinkling was neglected, as well as the nominal case, to highlight the significance of these response drivers.

**Figure 13:**
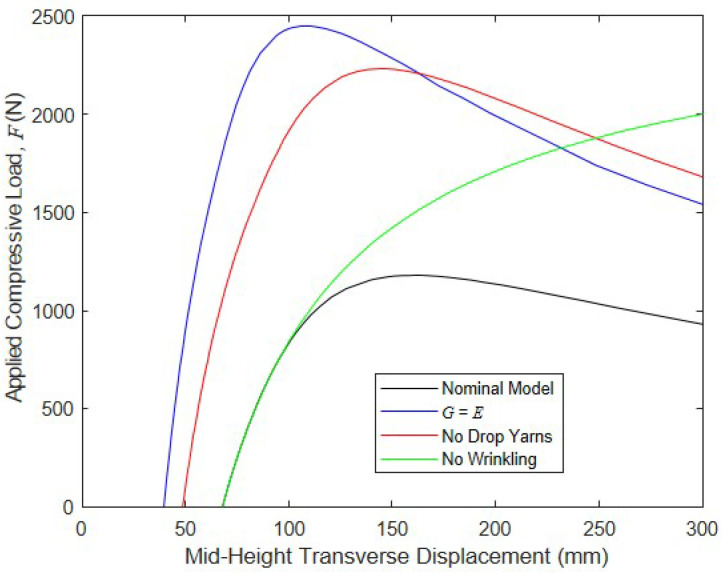
Impact of parameters on the buckling and post-buckling responses with a simultaneously applied transverse load.

The results of the analysis are shown in Figure 13. The first observation is that the application of load Q caused both significant initial transverse deflections when F= 0 that ranged from 40 to 68 mm for the four conditions. Further, comparing the results in Figure 13 with those of Figure 12 shows that the application of a constant Q dramatically decreased the peak axial loads in all cases. As with the case of pure buckling, using an artificially large value of G and ignoring the drop-stitch yarns dramatically increased the peak axial capacity relative to the nominal case, highlighting the importance of considering these effects. In contrast, in the case of pure buckling, however, wrinkling had a much more dramatic effect on the response: the nominal model had a peak axial capacity of 1180 N and exhibited a significant post-peak softening, whereas in the case in which the wrinkling was neglected, the model predicted that the panel would not buckle even at a transverse displacement of 300 mm, where it was still carrying an axial compressive load of 2000 N. Taken as a whole, these results reinforce the importance of considering all facets of the response when considering the combined transverse and compressive loading of drop-stitch panels.

One major contributor to the capacity that deserves further attention is the inflation pressure, p, for the panels subjected to both bending and compression. To this point, all simulations assumed a p= 68.9 kPa, which is a typical operating pressure for drop-stitch panels in bending-dominated applications. However, in References [24,25], the flexural response of drop-stitch panels was studied analytically and experimentally at pressures of 34.5 kPa, 69 kPa, and 103 kPa, with an increasing pressure increasing the panel’s stiffness and bending capacity. This was expected, since increasing p both directly increases the effective shear rigidity and will delay the onset of wrinkling, as discussed in Section 2. It must be noted that prior studies have also demonstrated an increase in the fabric moduli E and G as inflation pressure increases, which can be attributed to higher inflation pressures straightening the fiber tows and increasing the inter-tow friction [25]. However, the effect of the increasing moduli with the increasing p was not considered here, as its magnitude would depend on the particular fabric types used for the panel’s construction, and the current objective was to isolate the effect of p.

**Figure 14:**
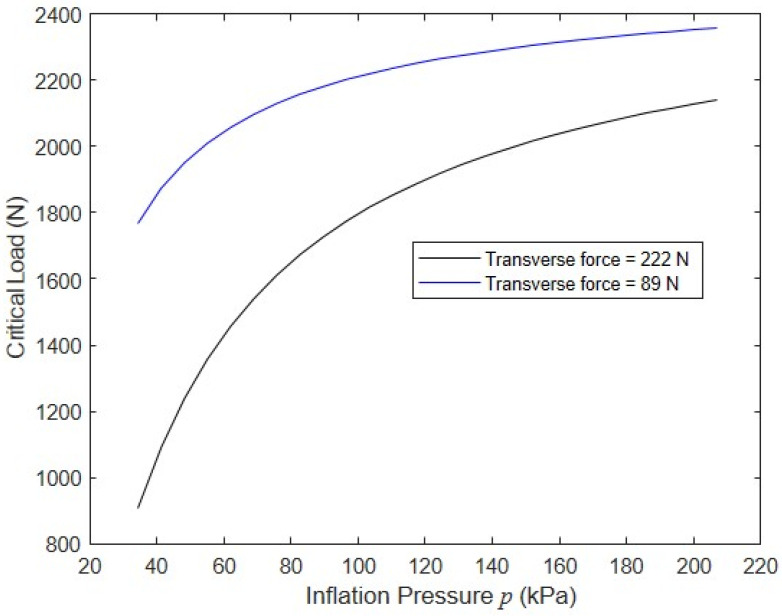
Impact of the transverse force and inflation pressure on the buckling and post-buckling responses.

To assess the impact of the inflation pressure, the nominal model was run with two values of a constant transverse load at mid-height, Q= 89 N and 222 N, while the corresponding axial buckling load F was determined for inflation pressures ranging from 34.5 kPa to 207 kPa. The larger value of Q = 222 N was below the wrinkling load of 328 N for the lowest pressure p= 34.5 kPa. Pressures exceeding the usual upper bound of 103 kPa were considered to provide insight into the response of panels constructed with new techniques that would allow for the use of higher pressures. (As a reference, braided fabric inflatable structural members with circular cross-sections can operate at inflation pressures of 412 kPa or higher [30]). The simulation results shown in Figure 14 clearly show that increasing the inflation pressure significantly increases the panel’s compressive capacity and that the relative increases were greatest for larger values of Q. This can be attributed to both the fact that increasing p delays the onset of wrinkling and that increasing p decreases the transverse deflection by increasing the effective shear stiffness.

## 5. Effect of Combined Snow and Wind Loading on the Model Structure

The material presented thus far has explored the response of isolated drop-stitch panels for simple cases of combined compression and transverse loading. In contrast, the objective of this section is to assess the feasibility of using drop-stitch panel walls as structural members in a practical application. Toward this end, the response of the drop-stitch panel walls of the model structure shown in Figure 1a to realistic loads caused by wind and snow, the two loadings typically governing the design of such structures, is explored here.

### Model and Loading

The model structure was assumed to have 2438 mm high walls made of multiple panels with a width of w=1219. The model structure’s overall width (horizontal wall-to-wall dimension) was taken to be 3658 mm. These three dimensions were chosen since they are typical in building construction in the United States. The wind load was taken as a uniform pressure, $p_{w}$, acting on the full 2438 mm high by 1219 mm wide panel, as prescribed by ASCE 7–10 [31], the specification governing design loads on buildings and other structures in the US. Treating the structure as an enclosed building with an exposure category C, as per the ASCE, and summing the contributions of the external positive and internal negative pressures, the relationship between the wind pressure, $p_{w}$ (N/m^2^), and wind velocity, v (m/s), as defined by the ASCE 7–10, reduces to Equation (19).

(19) $$p_{w}=0.4481v^{2}$$

The snow load pressure, $p_{s}$ (N/m^2^), was taken as a uniform gravity load acting downward on the horizontal projection of the roof. Gathering the loads, $p_{s}$ reduces to a single axial compressive load, $F_{s}=1.219\times 1.829{\times p}_{s}$, since half of the overall width of the structure contributes to $F_{s}$. The wall can then be analyzed as a pinned–pinned beam-column subjected to a uniform transverse load, $q_{w}=1.219\times 0.4481\times v^{2}$ (N/m) and compressive force $F_{s}$ (N).

**Figure 15:**
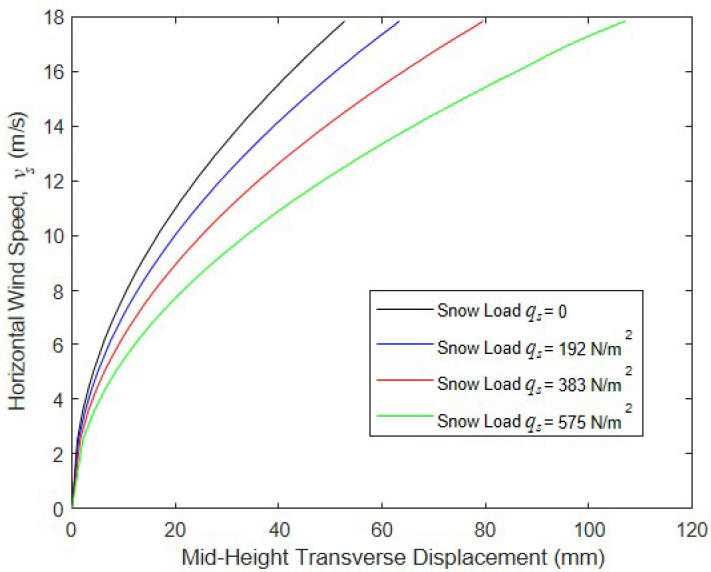
Wind speed vs. mid-height transverse displacement for various levels of $p_{s}$.

To assess the impact of the different levels of snow load, the model structure was analyzed for wind velocities ranging from 0 to 17.9 m/s and roof snow pressures of $p_{s}=$ 0, 192, 383, and 575 N/m^2^. In these initial simulations, inflation pressure p was fixed at 68.9 kPa and panel depth h= 102 mm. These values of wind speed and snow load are not extreme but could be expected to be encountered with some regularity across much of the northern portion of the continental United States and in other parts of the world. The average unit weight of new top snow (typically called “powder”) is approximately 1500 N/m^3^ [32], which implies that the upper snow load of 575 N/m^2^ corresponds to a depth of 38 cm. However, wet compacted snow can be three or more times denser than this [32], implying a depth of 13 cm or less to produce 575 N/m^2^. The results of these analyses are given in Figure 15, which clearly show a strong dependence of the wall response on the snow load. However, the maximum mid-height transverse displacement of the drop-stitch wall is 107 mm for the largest value of $p_{s}$, and no sign of instability is evident in this figure.

**Figure 16:**
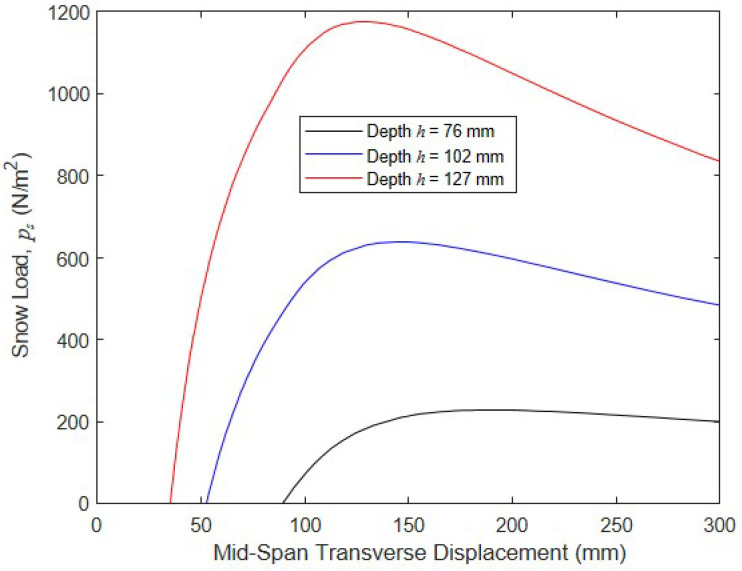
Wall buckling and post-buckling response for different values for the wall depth and the constant v= 17.9 m/s.

A critical design parameter for the wall was the panel depth, h: as h increased, the panel bending stiffness increased dramatically; the pressure resultant P and, thus, the effective shear rigidity increased; and the onset of the wrinkling and subsequent softening occurred at higher loads. To assess the impact of the wall depth and its impact on the ultimate wall capacity, a series of analyses were run where walls with h= 76, 102, and 127 mm were modeled while the wind speed, v, was fixed at 17.9 m/s, $p_{s}$ was increased until the wall collapsed, and the post-buckling response was tracked to transverse deformations of 300 mm. The results of these analyses are shown in Figure 16. The original wall with a depth of 102 mm carried a peak $p_{s}$ of 639 N/m^2^ at a corresponding transverse displacement of 144 mm, which is only 11% greater than the maximum value of $p_{s}$ of 575 N/m^2^ considered in the first set of analyses. Put another way, a 102 mm thick wall only has a factor of safety of 1.11 against collapse when subjected to a 17.9 m/s wind while carrying a snow load of 575 N/m^2^. However, increasing the panel depth by only 25 mm to 127 mm while keeping all other properties constant increased the maximum snow load capacity to 1176 N/m^2^, providing a very reasonable factor of safety against collapse of 2.05. Conversely, decreasing the wall depth to 76 mm reduced the wall snow load collapse pressure to 229 N/m^2^. These results clearly highlight the importance of the wall panel depth for this practical structural configuration.

**Figure 17:**
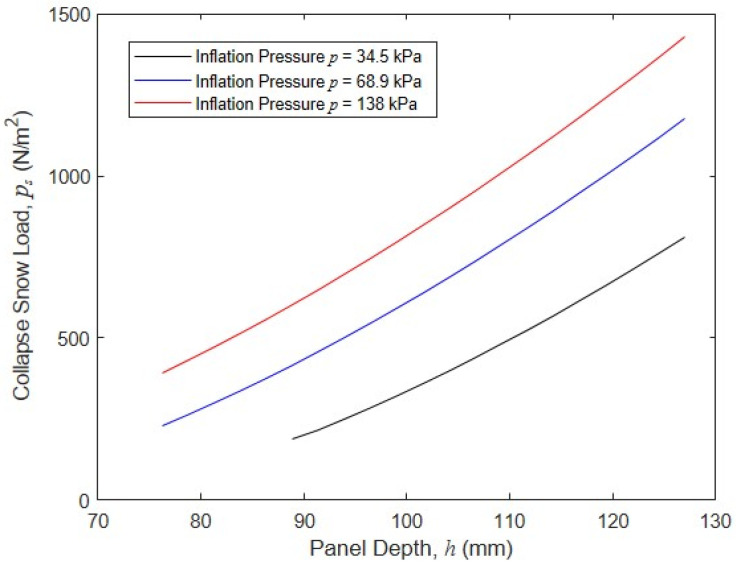
Wall collapse snow load as a function of the wall depth and pressure with a constant wind velocity of v= 17.9 m/s.

Another important design parameter is inflation pressure. To examine the significance of p and its interaction with h, a series of simulations were run where the effect of panel depth h on the collapse snow load was quantified for different values of p= 34.5 kPa, 68.9 kPa, and 138 kPa. In each simulation, the panel was subjected to a constant wind velocity of v= 17.9 m/s, and increasing snow load pressures $p_{s}$ were applied until the panel collapse load was reached. This process was repeated for panel depths ranging from 76 mm to 127 mm. The results of these simulations are shown in Figure 17, and indicate that increasing p in conjunction with h can markedly increase the panel collapse load. For example, for an intermediate panel depth of 102 mm, increasing p from 68.9 kPa to 138 kPa increased the collapse snow load pressure from 640 N/m^2^ to 847 N/m^2^, a change of 32%. In contrast, reducing p to 34.5 kPa results in a drop in the collapse snow load of 44% to 357 N. These simulations clearly show that both inflation pressure and panel height are important drivers of panel capacity and that their impacts are coupled.

## 6. Conclusions

This study examined the behavior and capacity of inflated fabric drop-stitch panels subjected to combined compression and bending using large deflection beam-based finite element analysis. The work reported here expands on significant prior research into the bending and compression of inflatable fabric members with circular cross-sections, as well as a smaller body of existing work on the bending response of drop-stitch panels. In addition to detailing basic panel mechanics in the context of straightforward loading conditions, a parametric study was conducted on a drop-stitch panel used as a wall element in a rapidly deployable shelter subject to realistic snow and wind loads. The research presented here supports the following major conclusions.

1. The wrinkling of the panel skin, *p* − *V* work, and deformations significantly impact the load–deflection response, capacity, and post-buckling behavior of drop-stitch panels used as beam-columns. Shear deformations significantly reduce the axial compressive capacity both with and without transverse bending. Wrinkling is more significant in the post-buckling regime for pure axial compression, but it can have a large impact on the compressive buckling load when transverse loads are simultaneously applied.
2. The drop-stitch yarns increase the panel skin bending stresses when a panel undergoes shear deformations, and significantly decrease a panel’s compressive capacity. This effect does not occur in typical inflatable fabric members with circular cross-sections.
3. The simulations of a drop-stitch panel used as a wall in a structure subjected to snow and wind loads highlight the importance of both inflation pressure and panel depth on wall capacity. The results show that drop-stitch panels with a reasonable thickness and inflation pressure have potential for use in conventional structural configurations.

In the future, experimental investigations on the buckling and post-buckling responses of drop-stitch panels loaded in compression and combined compression and bending should be conducted to verify the mechanics and modeling strategies presented here. In parallel, initial geometric imperfections and curvature that influence the stability need to be quantified. Future developments in drop-stitch panel manufacturing techniques should focus on reducing shear deformations and allowing for higher safe operating pressures, both of which can significantly increase the efficiency of drop-stitch panels required to support significant compression and bending in structural applications.

## Data Availability

Data will be provided by the author upon reasonable request.
